# In Vitro and In Silico Studies of Soluble Epoxide Hydrolase Inhibitors from the Roots of *Lycopus lucidus*

**DOI:** 10.3390/plants10020356

**Published:** 2021-02-13

**Authors:** Yoo Kyong Han, Ji Sun Lee, Seo Young Yang, Ki Yong Lee, Young Ho Kim

**Affiliations:** 1 College of Pharmacy, Korea University, Sejong 30019, Korea; yookyong05@korea.ac.kr; 2 College of Pharmacy, Chungnam National University, Daejeon 34134, Korea; rmthghks@naver.com (J.S.L.); syyang@cnu.ac.kr (S.Y.Y.)

**Keywords:** *Lycopus lucidus*, Labiatae, soluble epoxide hydrolase, molecular modelling

## Abstract

Soluble epoxide hydrolase (sEH) is an enzyme that is considered a potential therapeutic target in human cardiovascular disease. Triterpenes (**1**–**4**) and phenylpropanoids (**5**–**10**) were isolated from *Lycopus lucidus* to obtain sEH inhibitors through various chromatographic purificationtechniques. The isolated compounds were evaluated for their inhibitory activity against sEH, and methyl rosmarinate (**7**), martynoside (**8**), dimethyl lithospermate (**9**) and 9″ methyl lithospermate (**10**) showed remarkable inhibitory activity, with the IC_50_ values ranging from 10.6 ± 3.2 to 35.7 ± 2.1 µM. Kinetic analysis of these compounds revealed that **7**, **9** and **10** were competitive inhibitors bound to the active site, and **8** was the preferred mixed type inhibitor for allosteric sites. Additionally, molecular modeling has identified interacting catalytic residues and bindings between sEH and inhibitors. The results suggest that these compounds are potential candidates that can be used for further development in the prevention and treatment for cardiovascular risk.

## 1. Introduction

Soluble epoxide hydrolase (sEH) is an all-pervading enzyme in vertebrates that transforms epoxides to their corresponding diols [1]. Substrates contain a diversity of environmental constituents, including an epoxide group. Especially, epoxy fatty acids such as arachidonic acid, formed by cytochrome P450 (CYP) epoxygenases, are endogenous substrates for the sEH C-terminal domain [2,3]. CYP epoxygenases metabolize arachidonic acid to four types of regioisomeric epoxyeicosatrienoic acids (EETs), all of which are biologically active [4]. EETs are proposed to be required in many biological systems containing ion channel regulation, vascular resistance, mitogenesis, and cell signaling [5]. The EETs have been found to have some cardiovascular effects, which include anti-inflammatory properties, increasing renal blood flow, promoting sodium excretion, and preventing migration in vascular smooth muscle cells [6,7]. A role for EETs in modulating blood pressure and in the pathogenesis of essential hypertension and pregnancy-induced hypertension has also been suggested [5,8]. The EETs, however, are further metabolized by soluble and microsomal epoxide hydrolases to form the responding dihydroxyeicosatrienoic acids [9]. Then, the reactions result in diminished cardioprotective effects of EETs. Accordingly, the pharmacological inhibition of sEH prevents EET debasement and reinforces the renal and cardioprotective effects of these metabolites [10].

*Lycopus lucidus* Turcz. (Labiatae) is a traditional edible herb native to East Asia, and is a perennial plant which occurs mostly in low wetland areas in Europe, Asia, and North America [11,12]. The aerial parts of *L. lucidus* have been used in traditional and folk medicine for the treatment of cardiovascular diseases, inflammation, disorders of menstruation, and edema for hundreds of years [13,14]. Additionally, aqueous extract of the leaves of *L. lucidus* inhibits vascular inflammatory process induced by high glucose levels [15]. Many researchers have studied the composition of the aerial parts of *L. lucidus*, although there are few reports on research about the roots of *L. lucidus*. The roots of this plant are edible and medicinal parts [16]. Additionally, their shape is similar to silkworms. The roots of *L. lucidus*, called “small ginseng” in China, have been attracting increasing attention, and have been studied for bioactivities such as antioxidant, antitumor, hypolipidemic, anti-aging, and hypoglycemic effects [17,18]. 

In this study, we researched the various chemical constituents of *L. lucidus* and evaluated their sEH enzyme inhibitory activities. Among its components, potential inhibitors have been shown to interact between receptors and ligands through enzyme kinetics and molecular docking studies. Based on the results, this study suggests the potential contribution of ingredients to the pharmacological activity of *L. lucidus* and its potential activity in the treatment and prevention of cardiovascular diseases. 

## 2. Results and Discussion

### 2.1. Isolation and Identification

**Figure 1:**
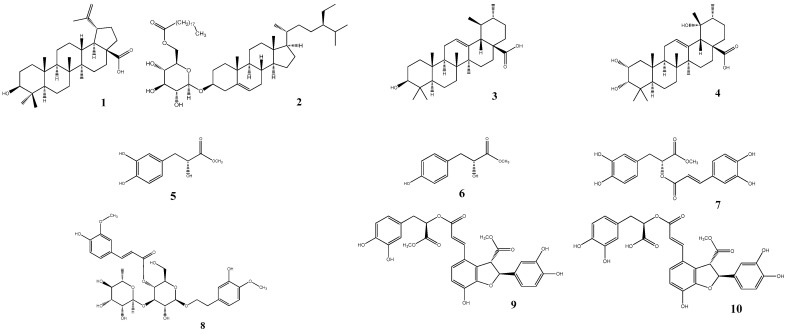
Structures of isolated compounds **1**–**10** from *L. lucidus*.

Methanol extract of *L. lucidus* roots was partitioned into ethyl acetate and distilled water fractions. Ten compounds (**1**–**10**) were isolated from the ethyl acetate fraction by silica gel and C18-reversed phase silica gel column chromatography (CC). The structures of compounds **1**–**10** were elucidated by comparing spectroscopic data with previously reported information in the literature. Ten compounds were identified: betulinic acid (**1**) [19], daucosterol 6’-O-nonadecanoate (**2**) [20], ursolic acid (**3**) [21], euscaphic acid (**4**) [22,23], (R)-(-)-oresbiusin A (**5**) [24], latifolicinin C (**6**) [25], methyl rosmarinate (**7**) [13], martynoside (**8**) [26], dimethyl lithospermate (**9**) [27,28], and 9″″-methyl lithospermate (**10**) [29] (Figure 1). 

### 2.2. sEH Inhibitory Activity

The ten isolated compounds **1**–**10** obtained in this study were evaluated for their sEH inhibitory activities. The tests were conducted using a multi plate system of the fluorometric photometer at excitation and emission wavelengths of 330 and 465 nm, respectively. A well-known potent sEH inhibitor, 12-(3-adamantan-1-yl-ureido)dodecanoic acid (AUDA), was used as a positive control (IC_50_ value of 2.0 ± 0.2 nM). To identify potential inhibitors of sEH, all compounds were measured at 100 µM concentration. As shown in Table 1, all isolated compounds (**1**–**10**) exhibited a wide range of sEH inhibitory activities. Among the evaluated compounds, compounds **1**, **3** and **6**–**10** showed over 50% inhibitory activity, and enzymatic assays were performed at various concentrations to determine the IC_50_ values of these compounds. Compounds **7**–**9** exhibited strong activity against sEH, with IC_50_ values of 16.8 ± 0.6, 19.5 ± 4.1, and 10.6 ± 3.2 µM, while compounds **1**, **3**, **6**, and **10** showed moderate activity, with IC_50_ values ranging from 35.7 ± 2.1 to 67.8 ± 3.2 µM. In this study is the first report of sEH inhibitory activity of compounds **6**, **7**, **9** and **10**. Compounds **1**, **3** and **8** have previously shown significant sEH inhibitory activity related to cardiovascular disease [30,31,32]. These sEH results showed that the phenylpropanoid derivatives exhibited stronger inhibitory activity than the triterpene compounds. It was also confirmed once again that, to the best of our knowledge, some of the phenolic derivatives explain their potential effects on sEH [33,34]. It was also suggested that diphenylpropanoid derivatives have stronger activity than phenylpropanoid derivatives through these results. 

### 2.3. Enzyme Kinetic on sEH

**Figure 2:**
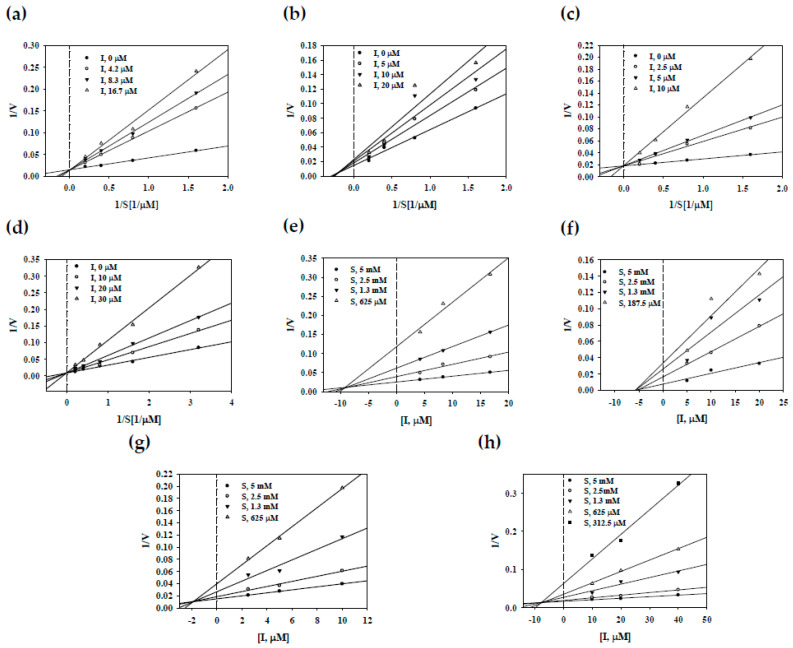
Lineweaver–Burk (**a**–**d**) and Dixon (**e**–**h**) plots of compounds **7**–**10** on sEH.

Enzyme kinetics studies were performed to confirm the nature of the interactions with enzymes for compounds **7**–**10**, which had IC_50_ values less than 40 µM. The enzymatic kinetics of these compounds (**7**–**10**) were determined by calculating the initial velocity of each inhibitor at various substrate concentrations (0.19–5 mM) (Figure 2). Compounds **7**, **9** and **10** were shown to have the same *V*_max_ value and different *K*_m_ values and were seen to intercept the positive *y*-axis; thus, they were identified as competitive inhibitors binding to the active site of sEH (Figure 2a,c,d). Compound **8** was observed to have various *V*_max_ and *K*_m_ values, and it intercepted the positive *y*-axis and the negative *x*-axis; thus, it was a mixed type (Figure 2b). This mixed type had bound to allosteric sites, a site different from the active site. In addition, the inhibitory constant values (*K*_i_) were calculated using the Dixon plot revised from the Lineweaver–Burk plot. As shown in Figure 2e–h and Table 1, the *K*_i_ values of compounds **7**–**10** were 9.2, 5.7, 1.9, and 7.6, respectively.

### 2.4. Molecular Docking Analysis 

**Figure 3:**
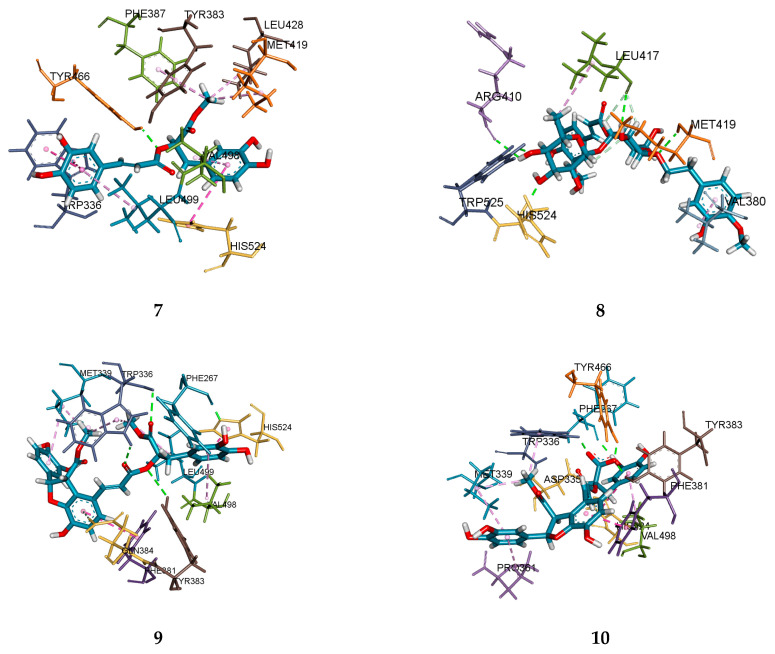
Molecular docking of compounds **7**–**10** with sEH (the green dotted lines are hydrogen bonds).

Molecular docking was used to simulate the interactional binding and poses between sEH and inhibitors subjected to enzyme kinetics. As shown in Figure 3 and Table 2, compounds **7**, **9** and **10** bound to the active site in sEH and acted as competitive inhibitors. The total scores of dockings for these compounds (**7**, **9** and **10**) were 8.60, 8.76, 5.93, respectively. Compound **7** had one hydrogen bond between the 9’ carboxyl group and Tyr466 (2.09 Å), and nine hydrophobic bonds with eight amino acids (Trp336, His524, Met419, Leu428, Tyr383, Phr387, Val498, Leu499). Compound **9** formed five hydrogen bonds with four amino acids (Phe267: 1.88 Å; Trp336: 2.36, 2.51 Å; Gln384: 2.71 Å; Tyr383: 3.06 Å) and nine hydrophobic interactions with five amino acids (His524, Phe381. Leu499, Met339, Val498). Three hydrogen bonds were formed between the carboxyl group 9, 9’ of compound **9** and Trp336 and Tyr383 of sEH, and one pi-donor hydrogen bond was formed between the aromatic ring and Gln384. Compound **10** had five hydrogen bonds (Tyr466: 1.70 Å; Tyr383: 1.85 Å; Trp336: 1.90 Å; Phe267: 1.98 Å; Asp335: 2.84 Å) and seven hydrophobic interactions (Trp336, Asp335, Phe381, His524, Met339, Val498, Pro361). The 9’ carboxyl group formed three hydrogen bonds with Trp336, Tyr383, and Tyr466, and there was one hydrogen bond between the 8’-H and Asp335. The 2’-OH of compounds **9** and **10** had a hydrogen bond with Phe267 in common. Although the position in the compound was different, it could be confirmed that they were catalytic amino acid residues because they commonly bound to amino acids Trp336, Tyr383, and Tyr466. Compound **8** preferentially bound to allosteric sites as a mixed type inhibitor, as revealed through enzymatic kinetics. Therefore, the active site as the binding site for this compound was excluded. The docking result was predicted to have the next higher overall score, similar to that of compound **8**. This compound formed seven hydrogen bonds with five amino acids (His524: 1.91 Å; Trp525: 1.99 Å; Met419: 2.31 Å; Leu417: 2.34, 2.75, 2.85 Å; Arg410: 2.58 Å) and two hydrophobic interactions with two amino acids (Leu417, Val380). It was confirmed that the sugar part of the compound mainly formed a hydrogen bond with His524, Trp525, Met419, Leu417, and Arg410, which are expected to be mainly amino acid residues of the allosteric site near the active site.

## 3. Materials and Methods

### 3.1. General Experimental Procedures

ESI–mass spectra were obtained on a Shimadzu LC MS-2020-EV. Nuclear magnetic resonance (NMR) spectra were obtained using a Bruker Fourier 300 (^1^H-300 MHz, ^13^C-75 MHz) and Jeol JNM-AL 400 (^1^H-400 MHz, ^13^C-100 MHz) spectrometer using TMS as an internal standard; chemical shifts were expressed as δ values. CC was carried out using 230–400 mesh silica gel (Kieselgel 60, Merck, Darmstadt, Germany) and RP-18 (ODS-A, 12nm, S-150 mM, YMC). The thin layer chromatography (TLC) was carried out glass plates pre-coated with silica gel 60 F_254_ and RP-18 F_254_ (20 × 20 cm, Merck). Compounds were visualized by dipping plates into 10% (v/v) H_2_SO_4_ reagent and then heated at 110 °C for 5–10 min. The solvents used for material separation, MeOH, *n*-hexane, CHCl_3_, EtOAc, *n*-BuOH, were purchased from SK chemical (Suwon, Gyeonggi-do, Korea). AUDA, sEH (10011669) and PHOME (10009134) were purchased from Cayman (Cayman, MI, USA.).

### 3.2. Plant Material

The dried roots of *L. lucidus* Turcz were purchased from Yakchomyoungga (Gyeonggi-do, Korea). A voucher specimen (CNU-16002) has been deposited at the Laboratory of Pharmacognosy, College of Pharmacy, Chungnam National University, Daejeon, Korea.

### 3.3. Extraction and Isolation

The dried roots of *L. lucidus* (2.7 kg) were extracted with methanol (15 L × 3) under reflux for 3 h. The methanol extract was then combined, filtered, and evaporated in a vacuum at 50 °C to yield 230.6 g of the extract. The residue (230.6 g) was suspended into distilled water (0.5 L), and the aqueous layer was partitioned with ethyl acetate (41.5 g).

The ethyl acetate layer was chromatographed over silica gel CC with n-hexane:EtOAc gradient (20:1 → 10:1), CHCl_3_:MeOH gradient (80:1 → 15:1) and CHCl_3_:MeOH:H_2_O gradient (7:1:0.1 → 3:1:0.1) to produce seven fractions (2A–2G) based on the TLC pattern. Fraction 2E was chromatographed on silica gel CC eluting with CHCl_3_:EtOAc gradient (50:1 → 7:1) and CHCl_3_:EtOAc:MeOH gradient (20:1:1 → 10:1:1) to obtain eight fractions (3A–3H) based on the TLC pattern. Among the eight fractions (3A–3H), compound **1** (561.8 mg) from the 3D fraction and **3** (585.8 mg) from the 3F fraction were separated into single spot on TLC, respectively. Fraction 3H was subjected to silica gel CC eluting with CHCl_3_:MeOH (35:1) to yield four fractions (4A–4D). Fraction 4C was chromatographed on C-18 CC eluting with MeOH:H_2_O gradient (2:1 → 4:1) to produce three fractions (5A–5C). Fraction 5A was subjected to C-18 CC eluting with (CH_3_)_2_CO:MeOH (1:6) to isolate compound **2** (9.9 mg) and to yield two fractions (6A–6B). Fraction 6A was subjected to C-18 CC eluting with MeOH:H_2_O (10:1) to isolate compound **4** (3.2 mg). Fraction 2G was chromatographed on C-18 CC eluting with (CH_3_)_2_CO:MeOH:H_2_O gradient (1:1:16 → 1:1:0.5) to produce seven fractions (10A–10G). Fraction 10A was chromatographed on silica gel CC using CHCl_3_:MeOH gradient (20:1 → 10:1) to isolate compound **5** (8.4 mg). Fraction 10B was subjected to silica gel CC using CHCl_3_:MeOH gradient (22:1 → 10:1) to isolate compound **6** (4.8 mg). Fraction 10D was chromatographed on silica gel CC eluting with CHCl_3_:MeOH gradient (20:1 → 12:1) and CHCl_3_:MeOH:H_2_O (4:1:0.1) to obtain compound **8** (4.1 mg). Fraction 10F was subjected to silica gel eluting with CHCl_3_:MeOH gradient (20:1 → 11:1) to obtain compound **7** (148.2 mg). Fraction 10G was chromatographed on silica gel CC eluting with CHCl_3_:MeOH gradient (20:1 → 15:1) to isolate compound **9** (17.2 mg). Fraction 10H was chromatographed on silica gel CC eluting with CHCl_3_:MeOH gradient (20:1 → 12:1) to isolate compound **10** (10.8 mg).

### 3.4. sEH Assay

The inhibitory activities on the sEH were measured according to the modified methods in previous papers [35,36]. PHOME was used as a substrate to examine the inhibitions of sEH, which was determined using a hydrolysis reaction of PHOME. sEH was diluted in Bis Tris–HCl buffer (25 mM containing 0.1 mg/mL BSA, pH 7.0) at 12.15 ng/mL. In a 96-well plate, 130 μL of recombinant human sEH, 20 μL of sample, and 50 μL of 40 mM PHOME were added. The absorbance was measured at excitation filter 330 nm and emission filter 465 nm for 30 min at an interval of 30 s after adding the last solution in a 96-well plate. All assays were performed in triplicate, using microplate reader (Tecan infinite F200, Grödig, Austria). Inhibitory activity (%) was calculated using the following equation:

(1) Inhibitory Activity (%)=100−[Δsample / Δcontrol]×100

where Δcontrol and Δsample are the changed fluorescence with and without the sample, respectively.

The concentration of the compound required for 50% inhibition of the substrate (IC_50_) values were calculated from a log concentration–inhibition curve, and AUDA was used as a positive control.

### 3.5. Molecular Docking Studies

Molecular docking studies was conducted by the Surflex–Dock method using SYBYL-X 2.1.1 (Tripos Ltd., St. Louis, MO, USA). The protein structure of human soluble epoxide hydrolase in complex with a synthetic inhibitor (PDB-ID: 3ANS) was retrieved from the Research Collaboratory for Structural Bioinformatics Protein Data Bank (RCSB PDB). The three-dimensional (3D) structures of the inhibitors were prepared by Chem3D Pro (version 12.0) and saved as mol files. The target regions of the proteins were prepared with the Tripos force field, and all the water molecules were removed. The binding region of the inhibitor was set to the active site or all protein. The docking was guided according to the Surflex preparation protocol in SYBYL-X 2.1.1. Discovery Studio 2019 Client (Biovia Co., San Diego, CA, USA) was used to visualize the positions of the ligand from the docked complex.

### 3.6. Statistical Analysis

The enzyme inhibition parameter IC_50_ was calculated by fitting hyperbola equations to the data using nonlinear regression of the plot using Sigma Plot (SPP Inc., Chicago, IL, USA.). The experimental results were performed in triplicate, and presented as the means ± SD.

## 4. Conclusions

In conclusion, *L. lucidus*, which has been used in cardiovascular disease in traditional drugs, was employed to find new sEH inhibitors. Among the triterpene (**1**–**4**) and phenylpropanoid (**5**–**10**) derivatives isolated from the roots of *L. lucidus*, compound **7**–**10** showed remarkable inhibitory activity against sEH. Enzyme kinetics and docking studies were conducted on these compounds. Taken together, these findings might explain the beneficial effects of *L. lucidus* and its constituents on cardiovascular diseases that need to be verified by further studies using in vivo models.

## Figures and Tables

**Table 1:** Inhibitory activity of compounds **1**–**10** on soluble epoxide hydrolase (sEH).

Compounds	sEH ^a^	
	IC_50_ (μM)	Type (Ki,μM)
**1**	67.8 ± 3.2	N.D.
**2**	N.D. ^c^	N.D.
**3**	42.1 ± 4.0	N.D.
**4**	N.D.	N.D.
**5**	N.D.	N.D.
**6**	52.0 ± 1.9	N.D.
**7**	16.8 ± 0.6	Competitive (9.2)
**8**	19.5 ± 4.1	Mixed (5.7)
**9**	10.6 ± 3.2	Competitive (1.9)
**10**	35.7 ± 2.1	Competitive (7.6)
**AUDA ^b^ (nM)**	2.0 ± 0.2	

^a^ All compounds were examined in a set of triplicated experiment. ^b^ Used as positive control. ^c^ Not determined.

**Table 2:** Inhibitory activity of compounds **1**–**10** on sEH.

Compounds	Docking Analysis (Total Score) ^a^	Hydrogen Bond	Hydrogen Bonds (Å)	Interacting Amino Acids Residues
**7**	8.6026	1	Tyr466 (2.09)	Tyr466, Trp336, His524, Met419, Leu428, Tyr383, Phr387, Val498, Leu499
**8**	7.6157	7	His524 (1.91), Trp525 (1.99), Met419 (2.31), Leu417 (2.34, 2.75, 2.85), Arg410 (2.58)	Met419, Trp525, His524, Leu417, Arg410, Val380
**9**	8.7626	5	Phe267 (1.88), Trp336 (2.36, 2.51), Gln384 (2.71), Tyr383 (3.06)	Trp336, Tyr383, Phe267, Gln384, His524, Phe381. Leu499, Met339, Val498
**10**	5.9275	5	Tyr466 (1.70), Tyr383 (1.85), Trp336(1.90), Phe267 (1.98), Asp335 (2.84)	Trp336, Tyr383, Tyr466, Phe267, Asp335, Phe381, His524, Met339, Val498, Pro361
**AUDA ^b^**	11.3220	4	Tyr343 (1.96), Tyr383 (1.82), Asp335(1.93, 2.83)	Tyr343, Tyr383, Asp335, Trp336, Met419, Pro268, Leu408, Trp525

^a^ Total score was expressed in −log10(*K_d_*)^2^ units to represent binding affinities. ^b^ Used as a positive control.

## References

[1] Fang X., Kaduce T.L., Weintraub N.L., Harmon S., Teesch L.M., Morisseau C., Thompson D.A., Hammock B.D., Spector A.A. (2001). Pathways of epoxyeicosatrienoic acid metabolism in endothelial cells implications for the vascular effects of soluble epoxide hydrolase inhibition. J. Biol. Chem..

[2] Newman J.W., Morisseau C., Harris T.R., Hammock B.D. (2003). The soluble epoxide hydrolase encoded by EPXH2 is a bifunctional enzyme with novel lipid phosphate phosphatase activity. Proc. Natl. Acad. Sci. USA.

[3] Schmelzer K.R., Kubala L., Newman J.W., Kim I.-H., Eiserich J.P., Hammock B.D. (2005). Soluble epoxide hydrolase is a therapeutic target for acute inflammation. Proc. Natl. Acad. Sci. USA.

[4] Seubert J.M., Sinal C.J., Graves J., DeGraff L.M., Bradbury J.A., Lee C.R., Goralski K., Carey M.A., Luria A., Newman J.W. (2006). Role of soluble epoxide hydrolase in postischemic recovery of heart contractile function. Circ. Res..

[5] Enayetallah A.E., French R.A., Thibodeau M.S., Grant D.F. (2004). Distribution of soluble epoxide hydrolase and of cytochrome P450 2C8, 2C9, and 2J2 in human tissues. J. Histochem. Cytochem..

[6] Imig J.D., Zhao X., Capdevila J.H., Morisseau C., Hammock B.D. (2002). Soluble epoxide hydrolase inhibition lowers arterial blood pressure in angiotensin II hypertension. Hypertension.

[7] Imig J.D. (2006). Cardiovascular therapeutic aspects of soluble epoxide hydrolase inhibitors. Cardiovasc. Drug Rev..

[8] Gupta N.C., Davis C.M., Nelson J.W., Young J.M., Alkayed N.J. (2012). Soluble epoxide hydrolase: Sex differences and role in endothelial cell survival. Arterioscler. Thromb. Vasc. Biol..

[9] Yu Z., Xu F., Huse L.M., Morisseau C., Draper A.J., Newman J.W., Parker C., Graham L., Engler M.M., Hammock B.D. (2000). Soluble epoxide hydrolase regulates hydrolysis of vasoactive epoxyeicosatrienoic acids. Circ. Res..

[10] Manhiani M., Quigley J.E., Knight S.F., Tasoobshirazi S., Moore T., Brands M.W., Hammock B.D., Imig J.D. (2009). Soluble epoxide hydrolase gene deletion attenuates renal injury and inflammation with DOCA-salt hypertension. Am. J. Physiol. Renal. Physiol..

[11] Yang X., Lv Y., Tian L., Zhao Y. (2010). Composition and systemic immune activity of the polysaccharides from an herbal tea (*Lycopus lucidus* Turcz). J. Agric. Food Chem..

[12] Moon H.-K., Kim Y.-C., Hong S.-P. (2013). Diagnostic characters and new populations of *Lycopus lucidus* var. *hirtus* (Lamiaceae). Kor. J. Plant Tax..

[13] Woo E.-R., Piao M.S. (2004). Antioxidative constituents from *Lycopus lucidus*. Arch. Pharm. Res..

[14] Li C., Li Z.-L., Wang T., Qian S.-H. (2014). Chemical Constituents of the Aerial Parts of *Lycopus lucidus* var. *hirtus*. Chem. Nat. Compd..

[15] Lee Y.J., Kang D.G., Kim J.S., Lee H.S. (2008). *Lycopus lucidus* inhibits high glucose-induced vascular inflammation in human umbilical vein endothelial cells. Vascul. Pharmacol..

[16] Lu Y.-H., Huang J.-H., Li Y.-C., Ma T.-T., Sang P., Wang W.-J., Gao C.-Y. (2015). Variation in nutritional compositions, antioxidant activity and microstructure of *Lycopus lucidus* Turcz. root at different harvest times. Food Chem..

[17] Yang X., Zhao Y., He N., Croft K.D. (2010). Isolation, characterization, and immunological effects of α-galacto-oligosaccharides from a new source, the herb *Lycopus lucidus* Turcz. J. Agric. Food Chem..

[18] Liang T., Fu Q., Li F., Zhou W., Xin H., Wang H., Jin Y., Liang X. (2015). Hydrophilic interaction liquid chromatography for the separation, purification, and quantification of raffinose family oligosaccharides from *Lycopus lucidus* Turcz. J. Sep. Sci..

[19] Cichewicz R.H., Kouzi S.A. (2004). Chemistry, biological activity, and chemotherapeutic potential of betulinic acid for the prevention and treatment of cancer and HIV infection. Med. Res. Rev..

[20] Ma X.-L., Lin W.-B., Zhang G.-L. (2009). Chemical Constituents of *Osmanthus yunnanensis*. Nat. Prod. Res. Dev..

[21] Taketa A.T., Breitmaier E., Schenkel E.P. (2004). Triterpenes and triterpenoidal glycosides from the fruits of *Ilex paraguariensis* (Maté). J. Braz. Chem. Soc..

[22] Tan J.-J., Tan C.-H., Chen Y.-L., Jiang S.-H., Zhu D.-Y. (2009). Chemical Constituents of *Clerodendranthus spicatus*. Nat. Prod. Res. Dev..

[23] Cheng J.-J., Zhang L.-J., Cheng H.-L., Chiou C.-T., Lee I.-J., Kuo Y.-H. (2010). Cytotoxic hexacyclic triterpene acids from *Euscaphis japonica*. J. Nat. Prod..

[24] Hwu J.R., Varadaraju T.G., Abd-Elazem I.S., Huang R.C.C. (2012). First Total Syntheses of Oresbiusins A and B, Their Antipodes, and Racemates: Configuration Revision and Anti-HIV Activity. Eur. J. Org. Chem..

[25] Zhou W., Xie H., Xu X., Liang Y., Wei X. (2014). Phenolic constituents from *Isodon lophanthoides* var. *graciliflorus* and their antioxidant and antibacterial activities. J. Funct. Foods.

[26] Yalcin F.N., Ersoez T., Akbay P., Çaliş İ., Dönmez A.A., STICHER O. (2003). Iridoid and Phenylpropanoid Glycosides from *Phlomis samia*, *P. monocephala* and *P. carica*. Turk. J. Chem..

[27] Wang L., Li X., Zhang S., Lu W., Liao S., Liu X., Shan L., Shen X., Jiang H., Zhang W. (2012). Natural products as a gold mine for selective matrix metalloproteinases inhibitors. Bioorg. Med. Chem..

[28] Zhang Z.-F., Peng Z.-G., Gao L., Dong B., Li J.-R., Li Z.-Y., Chen H.-S. (2008). Three new derivatives of anti-HIV-1 polyphenols isolated from *Salvia yunnanensis*. J. Asian Nat. Prod. Res..

[29] Thuong P.T., Kang K.W., Kim J.K., Seo D.B., Lee S.J., Kim S.H., Oh W.K. (2009). Lithospermic acid derivatives from *Lithospermum erythrorhizon* increased expression of serine palmitoyltransferase in human HaCaT cells. Bioorg. Med. Chem. Lett..

[30] Khanh P.N., Duc H.V., Huong T.T., Son N.T., Ha V.T., Van D.T., Tai B.H., Kim J.E., Jo A.R., Kim Y.H. (2016). Alkylphloroglucinol derivatives and triterpenoids with soluble epoxide hydrolase inhibitory activity from *Callistemon citrinus*. Fitoterapia.

[31] Cho I.S., Kim J.H., Lin Y., Su X.D., Kang J.S., Yang S.Y., Kim Y.H. (2020). Inhibitory Activity of Quercetin 3-O-Arabinofuranoside and 2-Oxopomolic Acid Derived from *Malus domestica* on Soluble Epoxide Hydrolase. Molecules.

[32] Tang H.-Y., Bai M.-M., Tian J.-M., Pescitelli G., Ivšić T., Huang X.-H., Lee H., Son Y.N., Kim J.H., Kim Y.H. (2016). Chemical components from the seeds of *Catalpa bungei* and their inhibitions of soluble epoxide hydrolase, cholinesterase and nuclear factor kappa B activities. RSC Adv..

[33] Li W., Kim J.H., Zhou W., Shim S.H., Ma J.Y., Kim Y.H. (2015). Soluble epoxide hydrolase inhibitory activity of phenolic components from the rhizomes and roots of *Gentiana scabra*. Biosci. Biotechnol. Biochem..

[34] Su X.D., Guo R.H., Yang S.Y., Kim Y.H., Kim Y.R. (2019). Anti-bacterial effects of components from *Sanguisorba officinalis* L. on *Vibrio vulnificus* and their soluble epoxide hydrolase inhibitory activity. Nat. Prod. Res..

[35] Kim J.H., Morgan A.M., Tai B.H., Van D.T., Cuong N.M., Kim Y.H. (2016). Inhibition of soluble epoxide hydrolase activity by compounds isolated from the aerial parts of *Glycosmis stenocarpa*. J. Enzyme Inhib. Med. Chem..

[36] Lee G.Y., Kim J.H., Choi S.-K., Kim Y.H. (2015). Constituents of the seeds of *Cassia tora* with inhibitory activity on soluble expoxide hydrolease. Bioorg. Med. Chem. Lett..

